# The Dual Blockade of the TIGIT and PD-1/PD-L1 Pathway as a New Hope for Ovarian Cancer Patients

**DOI:** 10.3390/cancers14235757

**Published:** 2022-11-23

**Authors:** Anna Pawłowska, Wiktoria Skiba, Dorota Suszczyk, Weronika Kuryło, Joanna Jakubowicz-Gil, Roman Paduch, Iwona Wertel

**Affiliations:** 1 Independent Laboratory of Cancer Diagnostics and Immunology, Medical University of Lublin, Chodźki 4a, 20-093 Lublin, Poland; 2 Students’ Scientific Association, Independent Laboratory of Cancer Diagnostics and Immunology, Medical University of Lublin, Chodźki 4a, 20-093 Lublin, Poland; 3 Department of Functional Anatomy and Cytobiology, Maria Curie-Skłodowska University, Akademicka 19, 20-033 Lublin, Poland; 4 Department of Virology and Immunology, Institute of Microbiology and Biotechnology, Faculty of Biology and Biotechnology, Maria Curie-Skłodowska University, Akademicka 19, 20-033 Lublin, Poland

**Keywords:** ovarian cancer, immunotherapy, programmed cell death pathway, TIGIT, dual blockade, clinical trials

## Abstract

**Simple Summary:**

Ovarian cancer (OC) is the most lethal gynecological malignancy with a five-year survival rate of 47%, followed by cervical cancer (66%), and uterine cancer (81%). Despite the success of immunotherapies based on the programmed cell death pathway (PD-1/PD-L1) in other solid cancers, the response of OC patients is low. The promising approach in OC treatment seems to be a combined therapy, including other immune checkpoints such as the TIGIT/CD155/DNAM-1 axis. The approach may trigger the synergistic effect, break the immunosuppression in the ovarian cancer tumor microenvironment, and enhance the expression of tumor antigens. The dual blockade stimulates the effector activity of T cells and NK cells, and redirects the immune system activity against tumor cells. The current understanding of the activity of both pathways, TIGIT/CD155/DNAM-1 and PD-1/PD-L1, as well as their synergistic action in OC, remains unclear.

**Abstract:**

The prognosis for ovarian cancer (OC) patients is poor and the five-year survival rate is only 47%. Immune checkpoints (ICPs) appear to be the potential targets in up-and-coming OC treatment. However, the response of OC patients to immunotherapy based on programmed cell death pathway (PD-1/PD-L1) inhibitors totals only 6–15%. The promising approach is a combined therapy, including other ICPs such as the T-cell immunoglobulin and ITIM domain/CD155/DNAX accessory molecule-1 (TIGIT/CD155/DNAM-1) axis. Preclinical studies in a murine model of colorectal cancer showed that the dual blockade of PD-1/PD-L1 and TIGIT led to remission in the whole studied group vs. the regression of the tumors with the blockade of a single pathway. The approach stimulates the effector activity of T cells and NK cells, and redirects the immune system activity against the tumor. The understanding of the synergistic action of the TIGIT and PD-1/PD-L1 blockade is, however, poor. Thus, the aim of this review is to summarize the current knowledge about the mode of action of the dual TIGIT and PD-1/PD-L1 blockade and its potential benefits for OC patients. Considering the positive impact of this combined therapy in malignancies, including lung and colorectal cancer, it appears to be a promising approach in OC treatment.

## 1. Ovarian Cancer—A Global Problem

**Figure 1:**
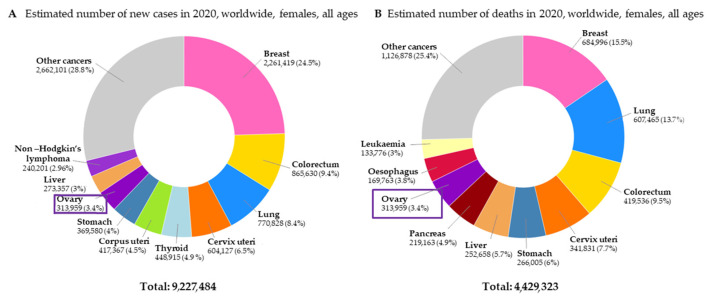
The estimated number of new cases of cancer (**A**) and deaths caused by cancer (**B**) among women worldwide [1].

Ovarian cancer (OC) is the third most common gynecological cancer and the most lethal gynecological malignancy. According to the latest World Health Organization (WHO) data, in 2020, as many as 313,959 women were diagnosed with OC and predictions concerning the near future are worrisome. There are predictions that in 2025, the number of new OC cases will increase to 823,315. Moreover, in 2020, the number of deaths caused by OC totaled 207,252 [1]. The estimated numbers of new cases of cancer and deaths caused by cancer among women worldwide are presented in Figure 1.

Despite the advances in the field of OC treatment and implementation of standard therapy, the prognosis for OC patients is poor, and the five-year survival rate is only 47% [2]. For comparison, the five-year survival rate for cervical cancer totals 66% and for uterine cancer 81% [3]. The standard treatment for OC patients includes primary debulking surgery and platinum plus taxane chemotherapy [4,5]. Respectively, in 2014 and 2018, the FDA approved biological drugs such as Olaparib (poly ADP-ribose polymerase inhibitor; PARPi) and bevacizumab (vascular endothelial growth factor inhibitor; VEGFi). In 2020, the FDA approved their combination (olaparib + bevacizumab) in treatment of BRCA-mutated OC [6]. The neoadjuvant chemotherapy followed by interval debulking surgery and subsequent chemotherapy as an alternative step is implemented in OC patients with medical complications, poor performance status, or visibly unresectable tumors [5]. The high mortality rate of the disease is caused by a lack of screening methods and diagnostic biomarkers in clinical practice and the high heterogeneity of the disease. Currently, the diagnosis of OC is mostly based on diagnostic imaging, laparoscopy, and the establishment of CA-125 in serum [7,8,9,10,11,12].

At the early stages of the disease, that is stages I and II of the International Federation of Gynecology and Obstetrics (FIGO) [13], the symptoms of OC are unspecific. As a result, OC is diagnosed mostly (<70%) at advanced stages (FIGO stages III and IV) when metastases occur and the five-year survival rate drops under 30% [9,10]. Approximately 70% of advanced OC patients have a recurrence and the disease becomes resistant to platinum-based chemotherapy [9,10,14]. It should be emphasized that OC at early stages is curable and the five-year survival rate is 90% at FIGO stage I and 70% at FIGO stage II [15]. Thus, it is important not only to develop novel effective therapy for OC patients but also to search for diagnostic biomarkers. 

Interactions in the tumor microenvironment (TME) appear to be the promising targets in up-and-coming OC treatment [16,17,18,19,20,21]. The immune system cells in TME are exposed to many signals that determine their immunophenotype and can manipulate their functions [22]. It is well established that cancer immunogenicity directly regulates immune evasion by various mechanisms, including immune checkpoints (ICPs), and is a critical factor in the prediction of response to ICPs inhibitors (ICIs) treatment [23,24].

Immunogenicity of OC depends on homologous recombination deficiency (HRD), including *BRCA-1/2* mutations. HRD tumors have an elevated neoantigens load and are infiltrated by T cells with high PD-1/PD-L1 expression enhancing the efficiency of ICIs-based immunotherapy. High-grade serous ovarian cancer (HGSOC) with *BRCA-1/2* mutations has a higher density of CD3^+^ and CD8^+^ tumor-infiltrating lymphocytes (TILs) in comparison to homologous recombination proficient OC [7,24,25]. The elevated density of TILs is also related to p53 mutations. This immunological signature is associated with prolonged overall survival (OS) compared with non-HRD OC types, which are less immunogenic and thus constitute more challenging targets for immunotherapies [7,23]. The hurdle in the establishment of OC immunogenicity is the heterogenicity of the disease at the molecular, genetic, and immunological levels [23].

## 2. Immune Checkpoints (ICPs) in Ovarian Cancer

In normal conditions, ICPs play an important role in maintaining homeostasis, which is a balance between immune response and tolerance of self-tissue in organisms, and a loss of their expression leads to autoimmune diseases [21,26,27,28,29]. However, the decreased effector activity of T cells and the suppressive activity of regulatory T cells (Tregs) are related to the modified expression of ICPs [26]. The predominant role in the inhibition of the effector activity of T cells in OC patients is played by the programmed cell death pathway, including programmed cell death receptor 1 (PD-1, CD279) mainly expressed on T cells, and programmed cell death ligands 1 and 2 (PD-L1/PD-L2). The PD-1 receptor belongs to the CD28 superfamily and plays an important role in both maintaining homeostasis and suppressing anticancer immune response. PD-L1 and PD-L2 are expressed on antigen-presenting cells (APCs) and various types of cancer cells, including lung cancer, kidney cancer, melanoma, and OC [21,30,31]. However, the expression of PD-L1 is wider and is also detected in T and B cells. It should be highlighted that PD-L2 has a 2–6-fold higher affinity to PD-1 [32]. In addition, it can directly bind to PD-1, while PD-L1 demands conformation changes. Nevertheless, the primary ligand for PD-1 is PD-L1 because its level is higher compared to PD-L2 [28,30,33,34,35,36]. The ligation of the PD-1 receptor and one of its ligands leads to anergy of T cells and their elimination via apoptosis [37]. The PD-1 activity does not lead to maintaining exhaustion but inhibits, at the primary step, the expansion of antigen-specific T cells [38].

Since 2011, when the Food and Drug Administration (FDA) approved monoclonal antibodies (mAbs) targeting against ICPs in melanoma therapy, ICIs have revolutionized cancer treatment. Considering the beneficial impact on the patient’s outcome, the immunotherapies based on anti-PD-1/PD-L1 mAbs were approved in other malignancies, including non-small-cell lung cancer (NSCLC), renal cancer, and hepatocellular carcinoma. However, the immunotherapy based on PD-1/PD-L1 inhibitors is not as effective as in other solid tumors and totals only 6–15% [16,17,18,19,20,21].

The immune evasion of OC cells is promoted by both PD-L1 and PD-L2. Thus, the boost of T-cells effector activity requires the complete inhibition of both ligands. The differences in the affinities of PD-L1 and PD-L2 to PD-1 suggest a competitive advantage of using anti-PD-L2 mAbs over the implementation of anti-PD-1 or anti-PD-L1 agents. Miao et al. [39] have shown that a high expression of PD-L2 is associated with poor clinical response on PD-1 and PD-L1 inhibitors [40]. A normal ovary tissue expresses an undetectable level of PD-L2, whereas OC tissue exhibits an increased level of PD-L2. Xue et al. [41] have observed the high expression of PD-L1 (43.04%) and PD-L2 (22.22%) in OC tissue. In addition, the PD-L2 expression is induced by signals provided from TME during tumor progression [39]. The PD-L1 expression on mononuclear cells from peripheral blood and peritoneal fluid of OC patients is increased in comparison to borderline and benign tumors [41]. The expression of these ligands is related to the FIGO stage and a high expression of PD-L1 or PD-L2 is associated with poor survival of OC patients. Moreover, patients with double negative tumors (PD-L1^-^ and PD-L2^-^) have a beneficial OS in comparison to patients with the expression of at least one ligand [40]. However, most of the ongoing clinical trials focus on the PD-1 or PD-L1 blockade while PD-L2 still remains poorly explored. The lack of studied agents targeted against PD-L2 is mainly caused by the assumption that the PD-L2 expression on cancer tissue is low and not significant [39]. 

The low to intermediate response of OC patients to immunotherapies based on the programmed cell death pathway blockade suggests that plenty of mechanisms relevant to the axis remain unclear. A combined therapy, including other ICPs, is a promising approach in OC treatment. The combination therapy triggers the synergistic effect, breaks the immunosuppression in the ovarian cancer TME, and enhances tumor antigens expression, yielding a better response [16,42].

## 3. The Net of TIGIT/CD155/DNAM-1 Signaling

Although T-cell activity is regulated mostly by the programmed cell death axis, other co-expressed ICPs have an indirect or direct impact on T-cell activity. The PD-1 is co-expressed with other ICPs, including T-cell immunoglobulin and ITIM domain (TIGIT; also called Vstm3, WUCAM, VSIG9), lymphocyte activation gene 3 (LAG-3), DNAX accessory molecule-1 (DNAM-1; CD226) and T-cell immunoglobulin and mucin domain-containing molecule-3 (TIM-3) in human cancers [29,43,44].

TIGIT was discovered, and for the first time described, in 2009 by Yu et al. The receptor is a negative regulator of natural killer (NK) cells and T cells, and it belongs to the nectin/poliovirus (PVR) family [45,46,47,48]. Nectins are the family of immunoglobulin-like cell adhesion molecules and play a crucial role in cell–cell adhesion, regulating the polarization of cells, their movement, survival and differentiation. The abnormal nectins expression is related to metastases and cancer progression [29,49,50,51].

Numerous recent studies have highlighted that the TIGIT/CD155/DNAM-1 axis plays a crucial role in immune response in both cancer and autoimmunity. Considering the sharing of the ligands by coinhibitory receptors, such as TIGIT, CD96, and costimulatory receptor DNAM-1, the TIGIT/CD155/DNAM-1 pathway appears analogous to the CTLA-4/CD28 axis [52,53,54,55]. In physiological conditions, the TIGIT/CD155/DNAM-1 axis plays a role in the maintenance of homeostasis and the loss of their function leads to autoimmune diseases such as multiple sclerosis, rheumatoid arthritis, and type 1 diabetes. The interactions between ligands and receptors of the TIGIT superfamily modulate immune system cell activity [20,26,56].

**Figure 2:**
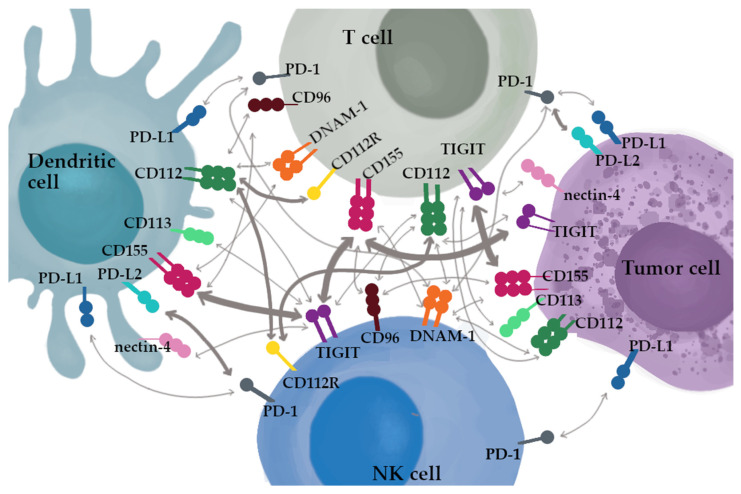
The net of TIGIT/CD155/DNAM-1 signaling. The thickness of the arrows represent the reported affinities.

Both PD-1 and TIGIT are considered a marker of exhaustion of CD8^+^ T cells [38]. The interactions between ligands and receptors in the TIGIT/CD155/DNAM-1 axis are greatly more complex than in the programmed cell death pathway. TIGIT is highly expressed on helper T cells, CD4^+^ Tregs, follicular CD4^+^ T cells, cytotoxic T cells (CD8^+^) and NK cells, while its expression is downregulated on naïve T cells [45]. The ligation of TIGIT and its ligand CD155 or CD112 lead to the inhibition of T-cell proliferation, production of cytokines by CD4^+^ T cells and suppression of NK cells cytotoxic activity [57]. TIGIT is expressed on tumor cells, including lung cancer, colorectal cancer, melanoma and OC [58,59,60,61,62,63,64]. The net of TIGIT/CD155/DNAM-1 signaling is presented in Figure 2.

The TIGIT/CD155/DNAM-1 pathway synergizes with the programmed cell death pathway [29,43,44]. The coverage of the TIGIT/CD155/DNAM-1 and PD-1/PD-L1/PD-L2 pathways was observed [38]. Banta et al. [38] have demonstrated a mechanistic account that the TIGIT/CD155/DNAM-1 axis and the PD-1/PD-L1/PD-L2 pathways share costimulatory receptor DNAM-1. In patients with NSCLC, it was observed that TIGIT and PD-1 regulated DNAM-1 activation by the synergistic mechanism both in vitro and in intact cells. PD-1 and TIGIT discretely regulate and suppress DNAM-1 activity. Thus, their dual blockade is required to completely restore DNAM-1 costimulatory signaling [38,65,66,67]. The PD-1 blockade is not sufficient to restore the activity of DNAM-1 because of the TIGIT presence. Similarly, the single TIGIT blockade is not optimal to reintroduce DNAM-1 signaling. These findings indicate why the dual blockade has more promising clinical results [38].

The suppression of immune response by TIGIT is caused by a few mechanisms. The first one is ITIM-dependent direct negative signaling. The next one is relevant to an increased production of IL-10, and a reduced production of IL-12 after the ligation of CD155 to TIGIT on dendritic cells (DCs). The immune response is also inhibited by competition between the ligands binding coinhibitory receptor TIGIT and costimulatory receptor DNAM-1, in TIGIT’s favor [68].

CD155 (also known as PVR, Tage4, Necl-5) has the highest affinity for TIGIT and is expressed on various types of cells, such as antigen-presenting cells (APCs), epithelial cells, fibroblasts, endothelial cells (in physiological conditions and tumors), T cells, B cells, and fibroblasts, and is upregulated by DNA damage in viral infections and during cancer [20,44,69]. It is well established that CD155 is highly expressed in various types of tumors, including lung cancer, glioblastoma, melanoma, colorectal cancer, hepatocellular carcinoma, pancreatic cancer, and OC [29,44,60,70,71,72,73,74]. The data available in the literature show that TIGIT indirectly inhibits T cells-dependent immune response by inducing the expression of CD155 on DCs [57]. As a result, the maturation of DCs is inhibited, and it increases the production of immunosuppressive IL-10 [57].

The CD112 (nectin-2, PVRL2) ligand was for the first time identified in 2016, exhibiting lower affinity to TIGIT than CD155 [29,45,75]. CD112 is expressed on DCs, monocytes, T cells, B cells, and endothelial cells [20]. In addition, CD112 is also overexpressed in many tumors, including leukemia, epithelial cancers, multiple myeloma, and OC. Binding the ligand to its receptor leads to the inhibition of T-cells effector activity and inhibits the NK cells-dependent cytotoxicity [29]. However, the main receptor for CD112 is not TIGIT, but CD112R (PVRIG) [44,74]. Both CD155 and CD112 play an indirect role in cancerogenesis by modulating the immune function via interacting with receptors expressed by immune cells, including DNAM-1, CD96, and TIGIT [60].

Another ligand for TIGIT is CD113 (PVRL3, nectin-3), which plays a crucial role in the suppression of T-cells activity [29,76] The ligand is overexpressed in the nervous system components, including the cerebral cortex and hippocampus. The data available in the literature highlight its role in synaptic abnormalities induced by stress. Thus, CD113 seems to be a potential target in the treatment of cognitive disorders related to stress [51].

While the latest report suggests that TIGIT also works with nectin-4 and TIGIT is its only receptor, the data are, unfortunately, limited [77,78]. Nectin-4 is mostly expressed during fetal development and its expression decreases in healthy adults. The activity of nectin-4 is related to cell functions including invasion, migration, proliferation, and differentiation. It is noteworthy that nectin-4 expression is increased in some cancer types, such as lung, bladder, and breast cancer (BC) and its higher expression is related to a poorer prognosis for cancer patients [44,76,77,79].

TIGIT enhances the immunosuppression by competing with inhibitory receptor CD96 (TACTILE) and costimulatory receptor, DNAM-1, to bind to CD155 and CD112. The primary ligand for CD96 is CD155 and its affinity is weaker than to TIGIT but stronger than to DNAM-1. Moreover, the CD96 expression is limited to immune system cells such as NK cells, NKT cells, and T cells (αβ and γδ). Moreover, CD96 mediates in NK cells’ adhesion to targets expressing CD155 [80]. The T-cell activation leads to increased CD96 expression; however, the CD96 role in T-cell function remains unclear [29,52,80,81].

DNAM-1 is one of the crucial regulators of Tcell biology and is expressed in T cells, NK cells, and monocytes. Its expression is negatively correlated with PD-1 expression on TILs [20,22,46,61,69]. Moreover, the absence of DNAM-1 inhibits the responsiveness of cytotoxic T cells to T-cell receptor (TCR) stimulation [22].

In contrast to TIGIT, DNAM-1 stimulates the cytotoxic activity of NK cells and T cells. The binding of CD112 or CD155 to DNAM-1 enhances the lysis of cancer cells by NK cells. However, CD155 has a higher affinity to TIGIT than to DNAM-1. The study conducted on Chinese hamster ovary cells has indicated that the affinity of CD155 to TIGIT is 30–100 times higher in comparison to DNAM-1. However, another study by Okumura has shown that the affinity of soluble CD155 (sCD155) to TIGIT is similar or even three times weaker than to DNAM-1 in humans and mice [47,54,82,83]. The binding of CD155 to TIGIT expressed on NK cells declines their cytotoxic capacity and production of IFNγ [44]. The effector capacity of T cells depends on the balance between the expression of TIGIT and DNAM-1 on their surface. Moreover, TIGIT enhances immunosuppression indirectly by blocking the costimulatory signaling of DNAM-1 by inhibiting the homodimerization of DNAM-1 [44]. DNAM-1 is downregulated in healthy ageing and decreased by CD155 on cytotoxic T cells, including CD8^+^PD-1^+^ TILs in various types of tumors. The binding of CD155 to DNAM-1 leads to the internalization and degradation of DNAM-1 in a proteasomal-dependent way. The activity of DNAM-1 is also suppressed by PD-1 receptor activity, and the inhibition of PD-1 restores the costimulatory signaling of DNAM-1 [29,44].

## 4. The Activity of TIGIT/CD155/DNAM-1 in Malignancies

Both the TIGIT/CD155/DNAM-1 axis and the programmed cell death pathway play role in T-cell exhaustion, and TIGIT and PD-1 are co-expressed on CD8^+^ T cells in human cancer [43]. In addition, TIGIT is the main receptor that causes the exhaustion of NK cells. The inhibition of immune response by TIGIT is based on delivering the inhibitory signals to NK cells and T cells, enhancing the suppressive activity of Tregs and inducing tolerogenic DCs via an increased production of IL-10 and a decreased production of IL-12 as a result of binding CD155 and TIGIT on the DCs surface [26,43,68,84,85,86]. The binding of CD155 or CD112 to TIGIT inhibits the T-cells effector activity and leads to cancer immune evasion [87]. In a murine model, TIGIT activity suppresses anticancer immune response by Tregs [88,89].The dual blockade of TIGIT and PD-1 induces cytokine production, as well as the proliferation and degranulation of TILs. Thus, the dual blockade of the TIGIT and PD-1/PD-L1 pathway seems to be prominent immunotherapy, especially for patients in whom the blockade of a single pathway has proven insufficient [84].

In melanoma, CD8^+^ TILs with decreased DNAM-1 expression and TIGIT upregulation mostly express also PD-1. The downregulation of DNAM-1 on NK cells and cytotoxic T cells of melanoma patients suggests that TIGIT and DNAM-1 play an antagonistic role in immunosuppression during cancer [26,46]. Both PD-1 (mainly on CD8^+^ T cells) and TIGIT (on CD8^+^ T cells, Tregs and NK cells) are expressed in over 70% of melanoma TMEs [43,84]. Simon et al. have demonstrated that the frequency of the circulating population of cytotoxic T cells with the co-expression of PD-1 and TIGIT is a potential factor in the prediction of the success of anti-PD-1 immunotherapy in melanoma patients [90]. Moreover, an increased ratio of the TIGIT/DNAM-1 expression on tumor-infiltrating Tregs is related to a high percentage of Tregs in melanoma patients treated with mAbs targeted against PD-1 and/or CTLA-4. It suggests that the ratio of TIGIT/DNAM-1 is a potential stability marker of Tregs [44]. Kurtulus et al. have shown that the dysfunctional immunophenotype of effector T cells is related to an increased production of IL-10 by TIGIT^+^ Tregs [57].

The expression of TIGIT in BC is related to highly aggressive subtypes, including basal-like BC subtype, triple negative BC, and HER2 positive BC or higher-grade tumor [91,92]. The study by Stamm et al. has demonstrated that the blocking of TIGIT or CD155 induces the lysis of BC cells via healthy donor peripheral blood mononuclear cells (HD-PBMCs) and cytokine-induced killer cells (CIKs) derived ex vivo from HD-PBMCs [92].

Similar to other effector cells of the immune system, the activity of NK cells is regulated by activating and inhibitory receptors, including TIGIT [43,93]. The toleration or elimination of the targets by NK cells depends on a net of reaching signals. Considering their nature, NK cells can recognize and kill cancer cells without inhibitory signals and a higher expression of activating ligands induced by cellular stress [94]. The interactions of DNAM-1 with its ligands are crucial for retaining the cytotoxic capacity of NK cells and eliminating cancer cells. The expression of DNAM-1 is downregulated on NK cells in myeloid leukemia [29].

Moreover, the upregulation of CD155 expression on tumor cells enhances the growth of cancer cells and their migration [95]. The increased CD155 expression is related to poor survival of patients with NSCLC [96], BC [97], osteosarcoma [98], and pancreatic cancer [71]. Similarly, the higher expression of CD96 is related to poor prognosis in pancreatic, lung, and breast cancer [51,99]. In contrast, the loss of CD155 on cancer cells facilitates the reduction of tumor growth and was found to lead to a beneficial response to anti-PD-1 agents in a murine tumor model [100]. Furthermore, the upregulation of the CD155 expression in metastatic melanoma is related to poor response to anti-PD-1 mAbs [44,60,70,71,72,73]. The increased CD155 expression on cancer cells leads to the proteasomal degradation of costimulatory receptor DNAM-1 on cytotoxic T cells, eventually resulting in the dysfunctional activity of CD8^+^ T cells in both human and mouse tumors [44]. Interestingly, the concentration of sCD155 in the serum of cancer patients, including those with BC, gynecological malignancies (including OC), gastrointestinal, and lung cancer, is increased compared to healthy blood donors [82,101].

## 5. The Activity of TIGIT/CD155/DNAM-1 in Ovarian Cancer

Taking into account the poor efficiency of standard treatment and relapse of the disease, adjuvant immunotherapy seems to be a promising complementary approach for OC patients [94]. Ovarian cancer is considered as immunogenic cancer because of the presence of tumor-infiltrating lymphocytes. Both TILs and NK cells play a crucial role in OC immunity and elimination of OC cells. However, OC is a highly heterogonic disease and the knowledge concerning the prognosis based on TILs is challenging. High CD3^+^ TILs are associated with beneficial OS at early stages of clear-cell and endometrioid OC. Similarly, CD8^+^ TILs in endometrioid OC are related to beneficial OS [102]. However, OC is generally marked by low to moderate TILs density [17,42]. Glennon et al. demonstrated that TIGIT is a potential prognostic marker for patients with HGSOC and its expression, similarly to PD-1 and PD-L1, is related with higher TILs density and beneficial prognosis [103]. Verhaak et al. have described four different gene-expression signatures in OC based on data from the Cancer Genome Atlas (TCGA), including proliferative, mesenchymal, differentiated, and immunoreactive types. The OC patients with immunoreactive types of tumors, marked by increased TILs levels, had beneficial survival rates [21,104]. The activity of NK cells in OC is modulated by TME including immunosuppressive cells, cytokines, and soluble factors. The increased percentage of NK cells in the peritoneal fluid was related to the beneficial survival of OC patients [94,105]. 

Data concerning the clinical implications of the TIGIT/CD155/DNAM-1 axis blockade, especially combined with the programmed cell death pathway, in OC patients are scant. Considering the efficiency of the dual blockade in animal models of other malignancies, the mechanism of its action should be elucidated.

TIGIT regulates the anticancer immune response via CD4^+^ Tregs which are related to tumor burden in OC patients. The dual blockade of TIGIT and PD-1/PD-L1 factors enhances the effector function of CD8^+^ T cells by synergistic action [57,106,107,108].

DNAM-1 is downregulated on NK cells in OC. Moreover, DNAM-1 is a crucial activating receptor of NK cells in OC, and the decreased expression of DNAM-1 in OC patients is related to the increased expression of CD155 in OC cells [94,109]. The data available in the literature shows that OC patients with lower expression of DNAM-1 on NK cells from PF have poorer survival than patients with higher DNAM-1 expression [110]. Peritoneal fluid soluble factors and cytokines have a strong influence on both the immunophenotype and distribution of NK cells present in that microenvironment [94]. The expression of DNAM-1 ligands is often upregulated on malignant cells. In OC patients, NK cells cytotoxicity is associated with an increased expression of CD155 [29].

Maas et al. have demonstrated that the TIGIT blockade enhances the responsiveness of NK cells in OC [94]. Similar results were obtained by Xu et al. [111] and Zhang et al. [112] who showed the beneficial influence of the TIGIT blockade on the antitumor activity of NK cells in BC and colon cancer, respectively. Thus, the novel approach to ICI immunotherapy in OC focuses not only on restoring the effector T-cell activity but also on increasing the anticancer activity of NK cells. 

Smazynski et al. have demonstrated that T cells from PF and HGSOC frequently co-express PD-1 and TIGIT. The TIGIT expression increases after a standard clinical protocol [60]. The authors [60] have demonstrated the presence of CD155 expression in TILs-negative HGSOC, while PD-1 expression is positively related to TILs. The soluble form of the CD155 ligand is also frequently overexpressed, which is related to higher tumor burden [60,113]. Moreover, the expression of costimulatory receptor DNAM-1 on TIGIT^+^ T cells was extremely reduced, which suggests that TIGIT regulates DNAM-1 expression. The increased TIGIT expression and the decreased DNAM-1 expression on TILs may explain the poor response to the PD-1/PD-L1 blockade in HGSOC patients [60].

Moreover, Xu et al. have shown that the increased level of CD113 is positively correlated with the FIGO stage and related to the poor OS of OC patients. Both invasion and migration of OC cells are stimulated by metalloproteinase 9 (MMP-9) and MMP-2, which increase the CD113 expression. Thus, CD113 is a promising diagnostic biomarker for OC patients [51].

In their study, Zong et al. [114] have demonstrated that the expression level of gene *C100rf54* that encodes VISTA (a PD-1 homolog) is positively associated with genes *TIM-3*, *TIGIT* and *PDCD1LG2* (encodes PD-L2). These genes encode proteins crucial for immune evasion in human cancers. VISTA is the ICP that suppress the effector activity of T cells via expression on APCs as a ligand or on T cells as a receptor. A preclinical study has demonstrated that the dual blockade of VISTA and PD-1 has a synergistic effect in a murine colon cancer model [115]. VISTA is also expressed in human cancers such as colorectal cancer, NSCLC, gastric cancer, and its expression is upregulated in endometrial and OC [114,116].

Preclinical studies indicate that TIGIT plays a significant role in immunosuppression in OC. Chen et al. have shown that in the OC murine model, TIGIT expression is higher in immune system cells, including Tregs. The TIGIT signaling blockade has been found to lead to the beneficial survival of mice with OC via decreasing Tregs function. It suggests that TIGIT can enhance the activity of Tregs, especially CD4^+^, and create an immunosuppression signature in OC [107]. Scientific studies confirm that the increased percentage of Tregs in PF is related to poor prognosis for OC patients [107,108,117]. In the OC murine model, it has been observed that the TIGIT blockade by mAbs leads to a reduced percentage of Tregs and a decrease in their suppressive activity that is related to a reduced production of IL-10 after anti-TIGIT mAbs implementation [107]. In addition, the result of the TIGIT blockade is an activation of tumor-activated CD8^+^ TILs. The TIGIT ligand, CD155, is highly expressed in HGSOC in a pattern familiar to PD-L1 expression [118]. Thus, the full understanding of the biological activity of the TIGIT/CD155/DNAM-1 axis and the programmed cell death pathway in OC leads to developing a novel approach to enhance the antitumor response of NK cells and T cells.

## 6. TIGIT-Based Immunotherapy

Immunological agents are a crucial approach in keeping in check the progression of various malignancies, including melanoma, renal, and lung cancer. Moreover, in 2018, Prof. James Allison and Prof. Tasuku Honjo were honored with the Nobel Prize in Physiology and Medicine for the discovery of negative regulators of immune response and their application in cancer therapy via inhibition of negative regulation of the immune system, respectively, CTLA-4 and programmed cell death pathway. Their findings have brought a great contribution to the field of tumor biology and a novel approach to cancer treatment [18,119].

The breaking of immunosuppression in the ovarian cancer TME seems to be a promising approach in OC treatment [120]. However, the insufficient effectiveness of immunotherapy based on programmed cell death inhibitors has forced scientists to search for new strategies to cure OC. There are two main mechanisms that determine the efficiency of ICIs in OC patients. The first critical variable is the density of TILs (CD3^+^, CD8^+^) in the OC TME. Hot tumors such as NSCLC and melanoma with high density of TILs in TME generally have high response rates to immunotherapies based on ICPs. However, cold tumors with low TILs density, including neuroblastoma and prostate cancer, poorly respond to this kind of treatment. OC is marked by low to intermediate TILs density, so OC patients nominally respond to ICIs. The next crucial factor that determines the success of ICIs in OC patients is tumor mutational burden (TMB). OC is characterized by low to intermediate TMB [17,42].

Another parameter that influences response to ICIs is mismatch repair (MMR), the malfunction of which leads to genome microsatellite instability (MSI). Cancers with high MSI produce tumor antigens with a 10–100 times higher TMB compared to tumors with microsatellite stability. This results in elevated immunogenicity and a higher response to immunotherapy based on ICPs. The most immunogenic histological type of OC is clear-cell OC because of a high MSI level and a high density of CD8^+^ TILs. Thus, ICIs in clear-cell OC are five-fold more efficient compared to other OC types [42].

The results of phase two clinical trial CITYSCAPE (NCT03563716) indicated that in patients with non-small cell lung cancer with high PD-L1 expression, the combination of tirogolumab and Tecentriq (atezolizumab; mAbs anti-PD-L1) vs. atezolizumab plus placebo, clinically improved the progression-free survival and the objective response rate (ORR; respectively, PFS median 5.4 vs. 3.6 months; ORR median 37.3% vs. 20.6%). The drug combination is well tolerated by patients and the safety profile is similar to that of Tecentriq implemented as a single agent [120,121,122]. The CITYSCAPE clinical trial was the first randomized study indicating that the simultaneous TIGIT and PD-L1 blockade stimulates anticancer activity and boosts the immune response [121,122,123]. Based on the results of the study, tiragolumab was the first anti-TIGIT mAbs that was granted breakthrough therapy designation (BTD) from the FDA [121,124]. The FDA approved anti-PD-1 (nivolumab, nembrolizumab) and anti PD-L1 (atezolizumab, durvalumab) mAbs for lung cancer treatment [125]. The main goal of novel immunotherapies is a restoration of CD8^+^ T cells-dependent antitumor immune response to eliminate tumor cells via immediate cytotoxicity and to generate anticancer immunological memory [44]. The advancement of NK cells-based immunotherapy mainly consists in assessing the NK cells immunophenotype, lytic activity, and ICP expression [29].

Despite the development of plenty of novel drugs based on ICPs inhibition, none of them, whether as a single factor or in combination with other factors, has shown significant activity in the potential clinical application in randomized clinical trials, except for mAbs targeted against TIGIT (tiragolumab) [68].

**Figure 3:**
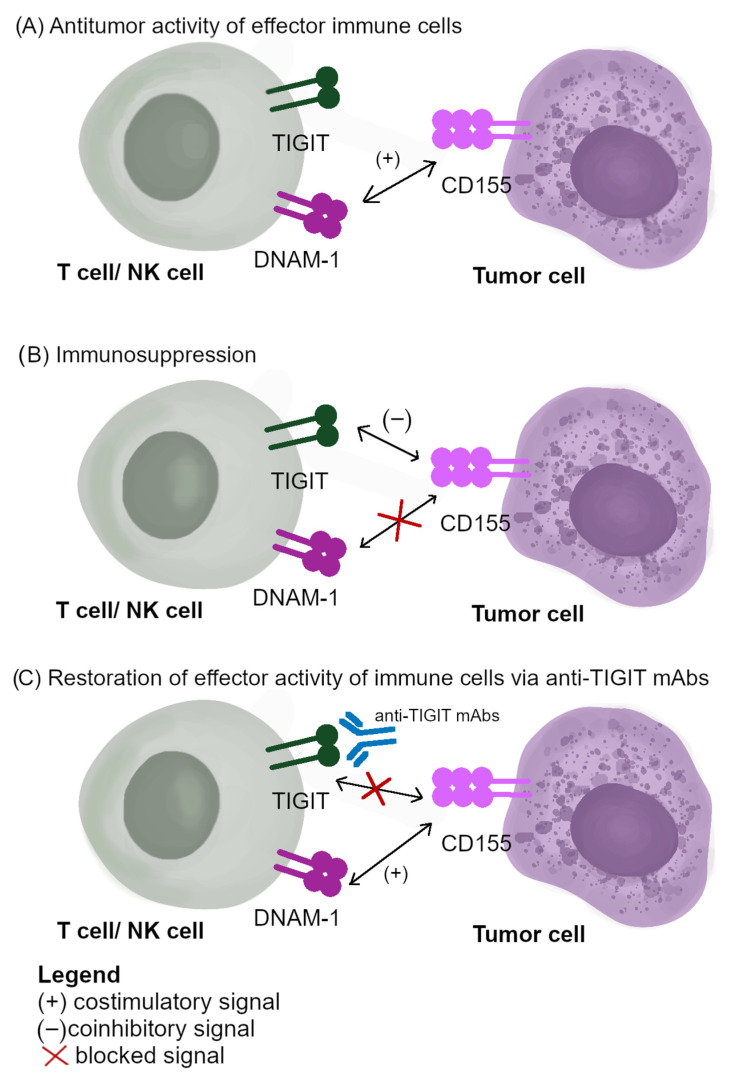
The role of TIGIT/CD155/DNAM-1 signaling in immunotherapies: (**A**) antitumor activity of effector immune cells; (**B**) immunosuppression; (**C**) the mode of action of anti-TIGIT mAbs.

Presently, a lot of mAbs targeted against TIGIT are tested in both preclinical and clinical trials to treat advanced and metastatic malignancies. The expression of PD-1 is limited to subsets of exhausted NK cells. In their study, Zhang et al. have shown that NK cells are crucial for obtaining a therapeutic effect of TIGIT inhibition or the dual of TIGIT and PD-1/PD-L1 pathway blockade [112]. Considering the complexity of TIGIT/CD155/DNAM-1, including sharing ligands, the prediction of the influence exerted by mAbs on the tumor blockade of TIGIT is challenging [77]. According to ClinicalTrials.gov, in 2022, 48 clinical trials have been launched, recruiting cancer patients to study the anti-TIGIT blockade in malignancies treatment. Most of them are currently at early implementation stages (phases 1–2) [126]. The main aim of early clinical trials is to establish in which group of cancer patients the implementation of single anti-TIGIT agents or in combination with anti-PD-1/PD-L1 mAbs will be beneficial [127]. There is growing evidence that the TIGIT blockade is a promising strategy that boosts the antitumor activity of NK cells even at advanced stages of the tumor [94]. Considering the role of the TIGIT/CD155/DNAM-1 pathway in malignancies progression, clinical trials are currently conducted using the mAbs targeted against these immune factors [128,129]. The results obtained in preclinical and clinical trials, including CITYSCOPE, suggest that the TIGIT blockade is the most promising ICPs inhibitor in the post-PD-1/PD-L1 era [26,68,121]. The role of the TIGIT/CD155/DNAM-1 pathway in immunotherapies and the mode of action of anti-TIGIT mAbs are presented in Figure 3.

Considering the predominant role of the TIGIT/CD155/DNAM-1 axis in the immunosuppression in tumors, including OC, the inhibition of the pathway in monotherapy or combined therapy, especially altogether with the programmed cell death pathway blockade, the ICP inhibitors are a potential target in cancer treatment, in particular in cold tumors [20]. Preclinical research has established that the TIGIT blockade works synergistically with the inhibitors of the programmed cell death pathway in combined therapy [46].

Promising results were obtained by implementing an anti-TIGIT agent combined with mAbs targeted against PD-1/PD_L1 in murine cancer models. In mouse models of colon carcinoma (CT26, MC38), BC (EMT6), and glioblastoma (GL261), the dual blockade caused fairly complete remission while the implementation of a single anti-TIGIT agent showed only limited effectiveness [26,62,85,130].

Dixon et al. [26] have demonstrated that a single TIGIT blockade in a murine model led to a modest inhibition of colon carcinoma growth, while the results of the PD-1 mAbs implementation alone were diverse, and a complete regression of the tumor was observed in only one animal out of a group of twelve mice. However, the combination of anti-TIGIT and anti-PD-1 agents resulted in a complete tumor regression in all tested mice (*n* = 12) [26]. The authors have also demonstrated that the double blockade of these pathways stimulates the effector activity of TILs by enhancement of TNF-α, IL-2, and IFN-γ secretion by CD8^+^ TILs, and increases the production of IFN-γ by CD4^+^ TILs [26]. Moreover, similar findings have been demonstrated by Chauvin et al. in melanoma [61], and by Ostroumov et al. in liver cancer [131].

In addition, DNAM-1 is required for the efficiency of the double blockade. Considering the role of DNAM-1 in the regulation of CD8^+^ mediated immune response, it plays an invaluable predictive role for estimating a success rate in ICIs treatment. In the dual TIGIT and PD-1 blockade, some differences between CD8^+^ T cells with high and low DNAM-1 expression were observed. The blockade by mAbs or the deficiency of DNAM-1 in tumor-bearing mice led to resistance to the double blockade. The observation indicates that DNAM-1 is indispensable for the dual blockade therapy [130].

The TIGIT blockade with mAbs leads to enhancing the antitumor response of effector T-cells regulated via NK cells. It also enhances the effectiveness of immunotherapy based on PD-1/PD-L1 inhibitors [112,132]. The double blockade is beneficial even in those cancer types that are resistant to PD-1/PD-L1 inhibitors. The dual blockade enhances the proliferation and production of cytokine, and the degranulation of CD8^+^ T cells [133]. Thus, the majority of clinical trials focus on combined therapy, including the inhibition of TIGIT and the programmed cell death pathway [44]. There are ongoing clinical trials that aim to establish the efficiency of the dual blockade of the TIGIT/CD155/DNAM-1 and PD-1/PD-L1 pathways in the treatment of such tumors as OC, esophageal cancer, and non-small-cell lung cancer [134]. The simultaneous implementation of anti-TIGIT and anti-PD-1 agents was beneficial in head and neck squamous cell carcinoma [135], glioblastoma [85], colorectal cancer [136], and renal cancer [137]. However, the synergetic mechanism of immunotherapy remains unclear [136].

The immunotherapy based on the TIGIT/CD155/DNAM-1 pathway is based on the competitive binding of CD155 with coinhibitory TIGIT or costimulatory DNAM-1, and it is a predominant mechanism of TIGIT-dependent immunosuppression. The blocking of TIGIT by mAbs leads to reversing the suppression [130]. However, the efficiency of the response to anti-TIGIT agents depends on the mAbs isotype (IgG1, IgG1 with lower affinity to FcγR, IgG4) and the prospect that CD155 binds to DNAM-1. The efficiency of the TIGIT blockade is lost if DNAM-1 is concurrently blocked [68]. The ongoing clinical trials using anti-TIGIT mAbs at advanced stages in cancer treatment are presented in Table 1.

In addition, immunotherapies combined with radiotherapy (RT) enhance the efficiency of treatment in many solid cancers and may constitute a new promising combination. The combination of anti-PD-L1 agents with RT stimulates the anticancer effect via CD8^+^ T cells-dependent mechanism that leads to the upregulation of PD-L1. Many clinical trials focus on the implementation of RT combined with ICIs in solid tumors, such as colorectal cancer, renal cancer, non-small cell lung cancer, sarcoma, and head and neck squamous cell carcinoma [126,144]. In their study, Grapin et al. have demonstrated that the combined therapy including RT and anti-PD-L1 and anti-TIGIT factors in a murine CT26 colon tumor model is beneficial in cancer treatment [144]. However, there is a necessity to conduct further studies to establish an optimal radiation pattern to successfully enhance the antitumor immune response in patients with particular types of cancer [144].

The development of bispecific antibodies (BsAbs) targeted at TIGIT and PD-L1 is another promising approach in cancer treatment. The BsAb is a combination of two tetravalent nanobodies (Nbs), i.e., anti-TIGIT Nb and anti-PD-L1 Fc-fusion Nb. The preclinical data in a murine model have demonstrated that the implementation of the BsAbs leads to increased anticancer activity in comparison to anti-PD-L1 mAbs. Its efficiency should be investigated in clinical trials [79,145].

Considering the success of ICIs, including anti-PD-1 and anti-PD-L1 agents, in the treatment of many solid cancers, including melanoma, lung, and kidney cancer, inhibitors of PD-1/PD-L1 appeared as a promising option for OC patients. However, the results obtained in clinical trials were disappointing [146,147]. Especially, monotherapies demonstrated only marginal outcomes in terms of response rate and survival improvement. Phase two clinical trials (KEYNOTE-100; NCT02674061) have indicated poor improvement in the OS (1.0625 fold) of OC patients after the implementation of pembrolizumab in monotherapy vs. chemotherapy (median OS, respectively, 18.7 vs. 17.6 months; PFS in both cohorts: 2.1 months) [148,149,150]. Considering that OC is a cold or warm tumor with a low to intermediate density of TILs, the combination of ICIs may improve the benefits of immunotherapy [17,151]. There are ongoing clinical trials that test anti-PD-1 (nivolumab, pembrolizumab) and anti-PD-L1 (avelumab, durvalumab, atezolizumab), both as single agents and in combination with PARP inhibitors (niraparib, olaparib) chemotherapy, antiangiogenic factors (bevacizumab) and other ICIs (ipilimumab, anti-TIGIT mAbs) [17]. Immunotherapies based on anti-PD-1 and anti-PD-L1 agents in OC patients were described in detail in our previous study [21].

According to ClinicalTrials.gov [126], in 2022, there are only four recruiting clinical trials applying the mAbs anti-TIGIT or anti-CD112R (COM701) in OC treatment, mainly in combination with anti-PD-1 agents (nivolumab). All of them are at the early implementation stages (phase 1 or 2) [126]. However, no results have been posted yet. It should be highlighted that OC is one of only few cancers in which ICPs inhibitors have not been accepted by FDA, either in combination or as monotherapy [17]. The ongoing clinical trials based on the TIGIT/CD155/DNAM-1 pathway in OC treatment are presented in Table 2.

Immunotherapy based on ICIs is well tolerated by cancer patients and rarely causes adverse events (AEs) including nonspecific symptoms, such as fatigue, fever, chillness, or immune-related adverse events (irAEs) as a result of immune response against self-tissue [17,156,157]. The most frequent irAEs are related to lungs, hepatitis, guts (diarrhea, colitis), skin (xerosis cutis, rash, and pruritus), and endocrine organs (thyroiditis). Most AEs occur in the first 12 weeks after ICIs implementation [17,156,157]. However, delayed AEs occur even several weeks or months after discontinuation of immunotherapy. Depending on the involved ICIs type, the irAEs are different but most of them are easy to manage with corticosteroids combined with immunosuppressants. The lethal side effects are rare and include myocarditis or acute renal failure [17,156,157]. In general, mAbs against TIGIT are well tolerated by patients in both monotherapy and in combination with PD-1 and PD-L1 inhibitors. The most frequent AEs include fatigue, pruritus, and, sporadically, diarrhea and anemia [79]. In treatment based on anti-PD-1 mAbs, the common irAEs is pneumonitis, while in patients treated with ipilimumab the frequent side effect is colitis [17,156,157]. The European Medicines Agency (EMA), in its pooled analysis, found the lowest irAEs incidence in patients treated with nivolumab as a single agent (78%) in comparison to ipilimumab alone (86%) and to a combination of nivolumab and ipilimumab (95%) [158]. ServeirAEs were found in 54% of patients treated with nivolumab and ipilimumab and five (0.7%) fatalities related to the treatment were reported, while patients treated with nivolumab as a single agent and ipilimumab alone showed lower irAEs incidence (14% and 27%, respectively) [156,158]. Another side effect of immunotherapy based on ICIs is hyperprogressive disease (HPD), which is related to the rapid cancer progression and tumor growth, resulting in shorter OS and PFS. The HPD incidence in tumors totals 4–29% [7,159]. However, in their study, Boland et al. have demonstrated that HPD concerned 46 out of 89 OC patients (51.6%) [160]. The occurrence of HPD in cancers suggests that immunotherapies based on ICPs inhibitors are harmful for some cancer patients. However, there are no factors to predict HPD and its mechanisms remain unclear [7,159].

## 7. Perspectives and Future Directions

In the past decade, various combinations of anti-PD-1/PD-L1 mAbs with other biological drugs (bevacizumab, olarparib) or chemotherapy were approved by the FDA in malignancies, including melanoma, cervical cancer, uterine cancer [79,148,161]. However, none of the anti-PD-1/PD-L1 mAbs were approved in OC treatment.

The main hurdle in efficient OC immunotherapy is connected with breaking the immunosuppressive TME [6]. The success of the dual TIGIT and PD-1/PD-L1 pathway blockade primarily depends on selecting the proper group of OC patients in whom the implementation of this kind of treatment would be beneficial. Thus, there is a necessity to identify the prognostic biomarkers of response in OC patients [79]. In addition, developing biomarkers would be helpful for researchers in combining the dual TIGIT and PD-1/PD-L1 pathway blockade with other agents, and in terms of minimizing its adverse effects and toxicity [6]. The complexity of the TIGIT/CD155/DNAM-1 axis and its mechanistic convergence with the PD-1/PD-L1/PD-L2 pathway is challenging in restoring the effector activity of both T cells and NK cells [66]. Thus, there is a necessity to develop combined therapies, including ICIs with PARPi and antiangiogenic factors. These combined therapies elevate the efficiency of immunotherapy via increasing TMB and the expression of tumor antigens and PD-L1 [17]. Further preclinical and clinical studies are necessary to understand the mechanisms underlying the synergistic action of the TIGIT/CD155/DNAM-1 and PD-1/PD-L1/PD-L1 pathways and to optimize immunotherapy [38].

The most challenging aspect of projecting immunotherapy for OC patients is the heterogeneity of the disease. There are no algorithms to select the proper OC patients group, in whom the immunotherapy would be beneficial. Considering the OC heterogeneity, it is hard to establish the correlation between the ICPs expression and response to the implemented therapy [16].

The next critical appearance in OC treatment is the hyperprogressive disease as an AE of immunotherapy. Distinguishing HPD and pseudoprogression is crucial for treatment continuation and for prolonging the OS of OC patients [162,163,164,165]. The pseudoprogression is the enhancement of primary tumor size or occurrence of new malignant infiltration in first weeks after implementation of immunotherapy. However, in pseudoprogression, tumor growth is related with its infiltration by immune cells, including T and B cells [166,167]. Thus, the establishment of pseudoprogression and HPD biomarkers for OC patients is predominantly to improve their clinical outcomes.

## 8. Conclusions

In the past decade, immunotherapies based on ICP inhibitors, mainly anti-PD-1/PD-L1 and anti-CTLA-4 mAbs, revolutionized the treatment of malignancies. However, their efficiency in some solid tumors, such as OC, is disappointing. Considering the modest efficiency of the currently used therapies and the lack of diagnostic biomarkers for OC patients, gaining insight into cancer surveillance and designing approaches to restore the antitumor capability of immune cells are highly needed.

Considering the latest report in the field of tumor immunology, the TIGIT/CD155/DNAM-1 pathway plays the predominant role in OC progression and interactions of its components with the programmed cell death pathway create strong immunosuppression in the ovarian cancer TME. The combined therapy, including the blockade of both axes, stimulates the effector activity of T cells and NK cells, and redirects the immune system activity against tumor cells. The current understanding of the activity of both pathways, TIGIT/CD155/DNAM-1 and PD-1/PD-L1, as well as their synergistic action, remains unclear. A full understanding of the role of TIGIT in OC immunity may help to project target therapies that will be beneficial in the disease.

## Figures and Tables

**Table 1:** The ongoing clinical trials using anti-TIGIT mAbs at advanced stages in cancer treatment.

Identifier	mABs	Blocked Immune Checkpoint	Additional Drugs	Condition	Phase	Company
NCT04746924 [138]	Ociperlimab	TIGIT	Tislelizumab Pembrolizumab	NSCLC	3	BeiGene
NCT04256421 [139]	Tiragolumab	TIGIT	Atezolizumab Carboplatin Etoposide	Small Cell Lung Cancer	3	Hoffmann -La Roche
NCT04294810 [140]	Tiragolumab	TIGIT	Atezolizumab	NSCLC	3	Hoffmann -La Roche
NCT04543617 [141]	Tiragolumab	TIGIT	Atezolizumab	Esophageal, Squamous Cell Carcinoma	3	Hoffmann -La Roche
NCT04736173 [142]	Domvanalimab	TIGIT	Zimberelimab Carboplatin Pemetrexed Paclitaxel	Squamous / Nonsquamous NSCLC	3	Arcus Biosciences, Inc.
NCT04866017 [143]	Ociperlimab	TIGIT	Tislelizumab Durvalumab Chemotherapy	NSCLC	3	BeiGene

**Table 2:** Ongoing clinical trials based on the TIGIT/CD155/DNAM-1 pathway in OC treatment.

Identifier	mABs	Blocked Immune Checkpoint	Additional Drugs	Phase	Company
NCT04354246 [152]	COM902	TIGIT	Monotherapy/ COM701	1	Compugen Ltd.
NCT04570839 [153]	BMS-986207	TIGIT	COM701 nivolumab	1/2	Compugen Ltd.
NCT05026606 [154]	Etigilimab	TIGIT	nivolumab	2	M.D. Anderson Cancer Center
NCT03667716 [155]	COM701	CD112R	nivolumab	1	Compugen Ltd.
